# Emerging Temporal Lobe Dysfunction in People at Clinical High Risk for Psychosis

**DOI:** 10.3389/fpsyt.2019.00298

**Published:** 2019-05-13

**Authors:** Paul Allen, Holly Moore, Cheryl M. Corcoran, James Gilleen, Petya Kozhuharova, Avi Reichenberg, Dolores Malaspina

**Affiliations:** ^1^Department of Psychology, University of Roehampton, London, United Kingdom; ^2^Department of Psychiatry, Icahn School of Medicine at Mount Sinai, New York, NY, United States; ^3^Department of Psychosis Studies, Institute of Psychiatry, Psychology and Neuroscience, King’s College London, London, United Kingdom; ^4^Department of Psychiatry, College of Physicians and Surgeons, Columbia University, New York, NY, United States; ^5^New York State Psychiatric Institute, University of Columbia, New York, NY, United States

**Keywords:** schizophrenia, temporal lobe, clinical high risk, hippocampus, dopamine, glutamate

## Abstract

Clinical high-risk (CHR) individuals have been increasingly utilized to investigate the prodromal phases of psychosis and progression to illness. Research has identified medial and lateral temporal lobe abnormalities in CHR individuals. Dysfunction in the medial temporal lobe, particularly the hippocampus, is linked to dysregulation of glutamate and dopamine *via* a hippocampal–striatal–midbrain network that may lead to aberrant signaling of salience underpinning the *formation of delusions*. Similarly, lateral temporal dysfunction may be linked to the *disorganized speech and language impairments* observed in the CHR stage. Here, we summarize the significance of these neurobiological findings in terms of emergent psychotic symptoms and conversion to psychosis in CHR populations. We propose key questions for future work with the aim to identify the neural mechanisms that underlie the development of psychosis.

## Introduction

Over the last few decades, early clinical interventions for people with psychosis have become more widespread, with an increasing clinical and research interest in identifying people presenting with early signs and symptoms of psychosis (1). Globally, a number of clinical services have been established that are aimed at preventative interventions. Such services identify people, usually young help-seeking individuals, experiencing prodromal symptoms characterized by attenuated psychotic symptoms (APS), including perceptual disturbances (subthreshold hallucinations) and overvalued ideas (subthreshold delusions), a brief psychotic episode, or exhibit a decline in social and occupational function coupled with familial risk (2). Individuals meeting one or more of these criteria are considered to be at a clinical high risk (CHR) for psychosis (2, 3), and around 20% will develop an onset of first-episode psychosis (FEP) within 1 to 2 years (with transition rates varying from 18% at 6-month follow-up to 36% after 3 years) (1). In addition to putative clinical benefits associated with early identification and intervention, research into early and prodromal phases of the illness may provide important information about the pathology of psychosis that is not confounded by long-term medication and/or illness chronicity. One approach is to longitudinally track neural changes using neuroimaging techniques such as magnetic resonance imaging (MRI) and positron emission tomography (PET). In 2002, Philips and colleagues (4) published the first neuroimaging study in a CHR population, reporting changes in hippocampal volume relative to age-matched healthy controls. This deficit neatly echoes the findings from meta-analyses in schizophrenia patients showing that the structure with greatest volume reduction compared to healthy individuals across the brain is the hippocampus (as well as amygdala and parahippocampus) (5, 6). Since then, several MRI studies [e.g., Refs. (7–10)] along with several PET studies [e.g., Refs. (11–14)] in CHR cohorts have been published, reporting a range of findings relating to brain function and connectivity, anatomy, and chemistry. While neuroimaging studies report neuroanatomical and neurofunctional changes across a range of cortical, subcortical, and cerebellar areas, neurobiological changes in two temporal lobe regions seem to feature prominently in the neurobiological basis of psychosis and the CHR state, namely, the medial temporal lobe (MTL) [e.g., Refs. (8, 15–20)] and the lateral temporal cortices [e.g., Refs. (21–24)]. Moreover, preclinical models [see Refs. (9, 25)] propose that neurobiological changes in temporal lobe regions may occur early in the developmental trajectory of psychosis and have a particular role in the development of attenuated psychotic symptoms (APS).

Furthermore, guided by work in animal models of schizophrenia (25–27) and findings from neuroimaging studies in patients with established schizophrenia, nascent neuroimaging research in CHR populations has identified the importance of an MTL and subcortical network involving the *hippocampus, midbrain, and striatum* (8, 9). Meanwhile, a separate body of neuroimaging work in CHR cohorts, again informed to a large extent by research in patients with established schizophrenia, has identified functional and anatomical alterations in the lateral temporal cortex and broader networks involving the frontal and parietal lobes (24, 28).

Given the role that progressive neurobiological changes in medial and lateral temporal lobe regions appear to play in the development of psychosis, we review evidence supporting dysfunction in temporal lobe-centered networks in people at CHR for psychosis and discuss how putative dysfunction in these regions and networks relates to the emergence of APS. We also consider how dysfunction and progressive changes in these regions track with conversion from the APS observed in CHR to first-episode psychosis (FEP).

## Clinical High-Risk Symptoms and the Onset of Psychosis

Prodromal symptoms are operationalized into criteria for a CHR state and are assessed using instruments such as the Comprehensive Assessment of At Risk Mental State (CAARMS) (29, 30) and the Structured Interview for Prodromal Symptoms/Scale of Prodromal Symptoms (SIPS/SOPS) (31). The criteria apply to young help-seeking individuals and require one of the following presentations: i) attenuated psychotic symptoms (APS), i.e., subthreshold delusions, hallucinations, and thought and language disturbances for which insight is preserved, ii) threshold psychotic symptoms that are brief and self-limiting, and iii) a significant decrease in functioning in the context of schizotypal personality disorder (SPD) or genetic risk for schizophrenia (32). Additional prodromal criteria may include subjective disturbances of cognitive processing and the perception of the self and the world (33, 34) and nonpsychotic symptoms such as anxiety and depression (35). More than 90% of CHR individuals present with APS, in particular attenuated delusions of unusual thought content and suspiciousness (36). While APS are almost always present in CHR individuals at clinical presentation, factor analytical studies of the CAARMS assessment instrument report a “disorganized symptoms” dimension that includes disorganized speech and thought, which is the best predictor of later conversion to FEP (37, 38), a finding also identified using the SIPS/SOPS (39–42).

Taken together, these symptom studies in CHR cohorts might suggest a pattern in which attenuated delusional ideation characterizes nearly almost all CHR cases, while disturbance in thought and language is more characteristic of those CHR cases that later develop psychosis. Is there any neuroimaging evidence to support this view, and what are the implications of these symptom patterns for emerging brain network dysfunction in CHR cohorts?

## Medial Temporal Lobe Network

While a range of neurobiological changes are identified in people with schizophrenia, including enlarged ventricles and volumetric reductions in the frontal lobes, thalamus, amygdala, hippocampus, and lateral temporal cortex (5, 43, 44), two neurobiological findings appear to be particularly robust. First, there are neuroanatomical and physiological alterations in the hippocampus and medial temporal lobe (MTL) (45–48) and second, elevated dopamine function in the midbrain and striatum [see Ref. (49) for review].

Based on decades of neuroimaging work describing anatomical, functional, and physiological changes in the hippocampus and MTL [e.g., Refs. (8, 10, 15, 17, 50)], in recent years, a number of researchers have developed a pathophysiological model that characterizes the progression of schizophrenia from the premorbid through the prodromal stages to syndromal psychosis. This model posits dysregulation of glutamate neurotransmission occurring in the CA1 region of the hippocampus that elevates neuronal activity, reflected in metabolism and blood flow, and in doing so elicits the APS emerging in the prodromal stage of schizophrenia (8, 19). As glutamate-driven dysregulation of the CA1 region of the hippocampus persists, dysfunction expands to projection fields within and external to the hippocampus and frontal cortex, leading to onset of threshold psychosis. According to this heuristic, as the illness progresses, an atrophic process ensues in which the neuropil of hippocampal cells is reduced, compromising numbers of interneurons, and leading to the volumetric reduction in the MTL and other regions observed in patients with schizophrenia (8, 19).

In line with this model, there is evidence from a number of neuroimaging studies of reduced hippocampal gray matter in CHR cohorts. Cross-sectional studies comparing CHR subjects to healthy controls have reported reduced gray matter volume in the hippocampal and surrounding MTL (4, 21, 51, 52), altered hippocampal function during word recognition (16), and elevated resting hippocampal perfusion (18, 53). Furthermore, prospective and longitudinal studies that include CHR subgroups who develop psychosis subsequent to scanning show that hippocampal volume, function, and perfusion changes can predict conversion to psychosis in CHR cohorts (8, 10, 15, 50, 54). However, it must be noted that there are negative studies too; see meta-analysis by Ref. (55). Within the MTL, reductions in volume and altered function have been localized to the anterior part of the hippocampus/parahippocampal gyrus (15, 50). A study by Schobel and colleagues (8) found that resting regional cerebral blood volume (CBV), a measure of metabolism and neuronal activity, was increased in the CA1 region of the hippocampus at baseline in CHR subjects who later developed psychosis. Moreover, longitudinal follow-up in this CHR cohort showed that the onset of psychosis was associated with a progressive increase in resting CBV in the anterior hippocampal subiculum that tracked with gray matter volume reduction in the same MTL region (8, 56).

That CA1 CBV is elevated in CHR *prior* to psychosis onset, and hippocampal subiculum CBV is elevated *after* onset, points to the progressive nature of hippocampal deficits in CHR that are associated with psychosis—and also offers a locus of enquiry as to how these two separate hippocampal regions may additively or differentially contribute to psychosis development in CHR. It is noteworthy that the subiculum is the primary output structure of the hippocampus (57), and also that the nucleus accumbens/ventral striatum receives its strongest excitatory input from the ventral subiculum—consistent with the central role of the ventral hippocampus in driving dopaminergic changes in the rodent model discussed below (25). The model proposed in the study by Schobel and colleagues (8) also posits that changes in hippocampal CBV during this pathophysiological process are driven by glutamate dysregulation. There is substantial evidence that glutamate is altered in schizophrenia and psychosis risk states (58–61). More recently, a prospective study by Bossong and colleagues (20) reports increased hippocampal glutamate levels in CHR subjects who later developed psychosis relative to CHR subjects who did not become psychotic. Additionally, ketamine, an N-methyl-D-aspartate (NMDA) receptor antagonist used as a model of schizophrenia, specifically raises CBV and glutamate levels in both CA1 and subiculum subregions of mouse hippocampus (8). By contrast, glutamate levels are generally *reduced* in medicated schizophrenia patients (62), together, suggesting that increased glutamate levels in the hippocampus may be more closely tied to the development and/or presence of symptoms rather than of a diagnosis of schizophrenia per se.

**Figure 1:**
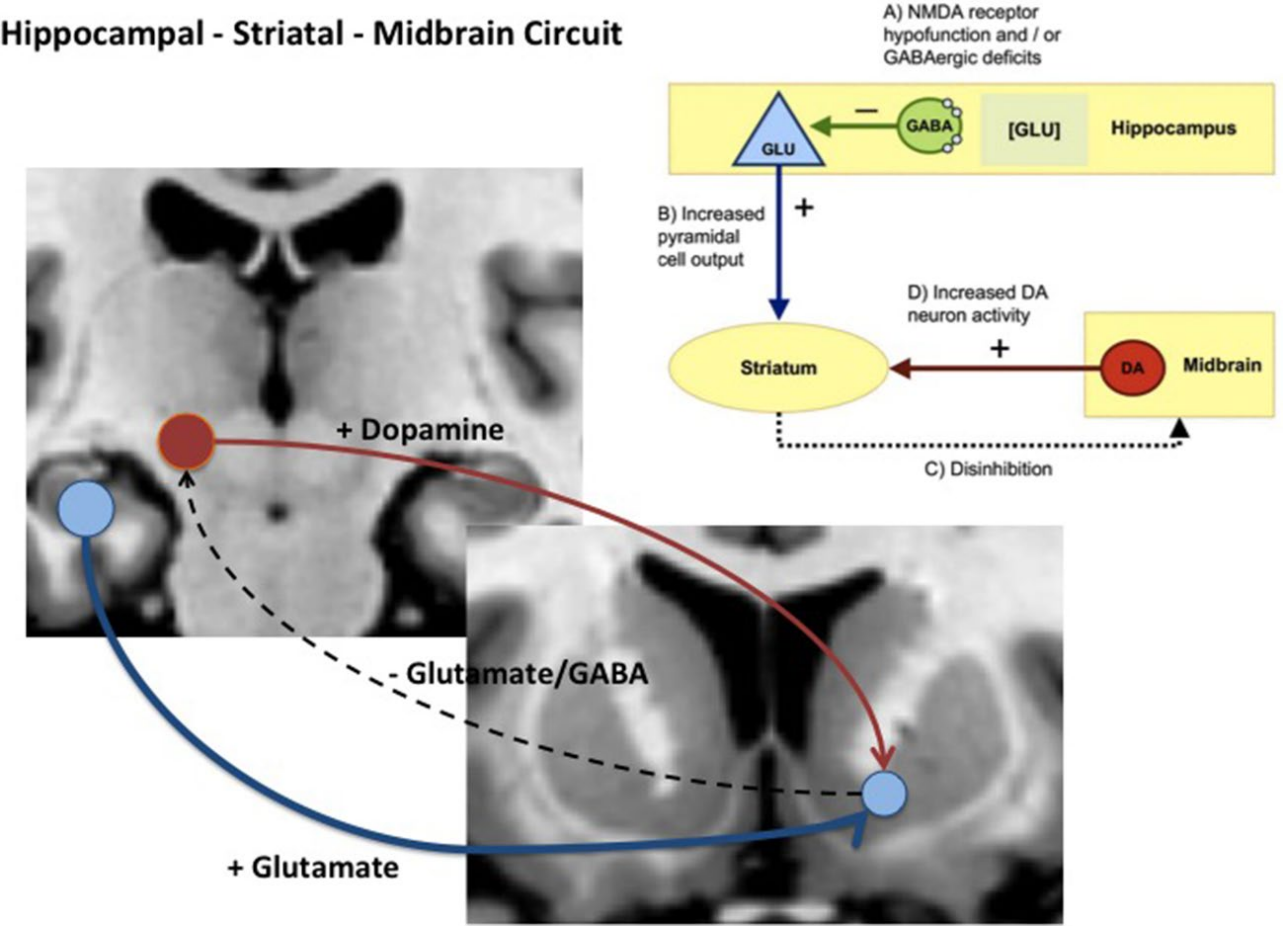
Diagram showing hippocampal midbrain striatal circuit involved in the regulation of striatal dopamine *via* glutamatergic, GABAergic projections (+) = excitatory pathway and (−) = inhibitory pathway. In schizophrenia and clinical high-risk (CHR) states, it is hypothesized that increased glutamatergic output from the hippocampal subiculum to the ventral striatum (blue pathway) reduces inhibition *via* glutamatergic and GABAergic pathways that ultimately drives ventral tegmental area (VTA) dopamine cells and dopamine release back to the striatum (red pathway).

Given these findings, how might glutamate-driven dysregulation of hippocampal or MTL physiology relate to elevated striatal and midbrain dopamine? Experimental work in rodents illustrates how changes in these two neurotransmitter systems might be linked and contribute to the development of psychosis. Studies in rats show that lesions or local glutamate receptor modulation activation of the ventral hippocampus leads to changes in the activity of midbrain Dopamine (DA) neurons and striatal DA release [e.g., Refs. (63, 64)]. Subsequently, several rodent disease models developed with the aim of recapitulating schizophrenia-relevant developmental neuropathology have supported strong links between hippocampal pathology, altered basal hippocampal activity, and dysregulation of midbrain dopamine neurons. One of the earliest of these models showed that bilateral excitotoxic lesions of the ventral hippocampus on postnatal day 7 in the rat altered excitatory activity in the hippocampus and produced changes to striatal dopamine markers (65, 66). In another well-characterized model, the MAM E17 rat, perturbation of neurodevelopment by administration of methylazoxymethanol acetate (MAM) to pregnant rats on embryonic day (E) 17 was associated with a number of histological, neurophysiological, and cognitive/behavioral deficits in the offspring that are analogous to those observed in some schizophrenia patients (25, 26). The most prominent histological abnormalities in the MAM E17 model appear in the hippocampus, including a reduction in the thickness and deficit in parvalbumin-expressing (PV+) gamma-Aminobutyric acid (GABA) ergic inhibitory interneurons. The MAM E17 model has highlighted the potential role of a *hippocampal–midbrain–striatal circuit* in the development of striatal dopamine dysregulation that, in turn, could drive the emergence of APS and psychosis. In this model, midbrain dopamine neuron activity and striatal dopamine levels are elevated (25, 26, 67), possibly as a consequence of aberrant hippocampal drive of ventral striatal projections (25, 26, 67) (see **Figure 1**). Indeed, inactivation of ventral hippocampus in MAM mice completely reversed the elevated dopamine activity (68). Of note, the elevation of ventral hippocampal activity raises not only tonic dopamine, but further, aberrant responsivity of the dopamine system *phasically* (68).

Complementary work in a genetic mouse model of a selective PV+ interneuron deficit in the hippocampus further supports a role for hippocampal dysregulation of striatal DA inputs (69). Deletion of the *cyclin D2* gene during embryonic development leads to a reduction in PV+ interneurons destined for the hippocampus. As adults, these mice show a selective, partial (40–50%) reduction in hippocampal PV+ interneuron density and decreased hippocampal thickness. Application of gadodiamide-enhanced contrast MRI to this model showed increased resting CBV in the hippocampus, similar to the imaging phenotype observed in CHR cases that convert to psychosis within 2 years (8). Neurophysiological studies show that, in adulthood, *cyclin D2* null mice show a deficit in inhibitory synaptic inputs onto hippocampal projection neurons, which is concurrent with excessive activity in midbrain/ventral tegmental area (VTA) dopamine neurons. Moving beyond descriptive and correlational studies, a seminal stem-cell transplant study in the *cyclin D2* null mouse and MAM E18 rat established a causal link between hippocampal disinhibition and excess activity of midbrain dopamine neurons. Specifically, restoring/supplementing GABAergic interneuron numbers in the hippocampus in adulthood through transplantation of PV+ interneuron precursor cells from the embryonic medial ganglionic eminence in these models, reduces metabolic activity in the hippocampus (69) and normalizes both the spike activity of the midbrain DA neurons and behaviors mediated by striatal DA transmission (70).

Consistent with the above animal models of schizophrenia highlighting abnormal hippocampal disinhibition, Allen and colleagues (18) reported increased resting cerebral blood flow (rCBF) across hippocampal, striatal, and midbrain regions in a CHR cohort relative to healthy controls [using an magnetic resonance (MR) perfusion measure known as arterial spin labeling] (71), a finding largely replicated in a second independent CHR cohort (53). Further, longitudinal analysis showed that normalization of hippocampal rCBF tracked with clinical improvement of symptoms in the CHR cohort, while elevated hippocampal rCBF persisted in those who remained symptomatic or developed psychosis (18).

To summarize, guided by work in experimental animals, the available evidence in humans, although limited, points toward emerging network dysfunction in a hippocampal–striatal–midbrain network that is driven by glutamate dysregulation resulting in disrupted hippocampal physiology, function, and eventually reduced volume. Furthermore, increased hippocampal neural activity leads to dysregulation of striatal–midbrain dopamine signaling. How dysfunction in this network relates to APS and conversion to FEP is discussed in more detail later (section Discussion: Emerging Temporal Lobe Dysfunction in Clinical High-Risk Populations, Attenuated Symptoms, and Transition to Psychosis). Currently however, most clinical studies investigate only one neurobiological component of the preclinical models discussed above. Clearly, to test preclinical models more rigorously, there is a need for more multimodal neuroimaging work that can better integrate information about neurochemical, physiological, functional, and anatomical factors.

## Lateral Temporal Lobe Network

**Figure 2:**
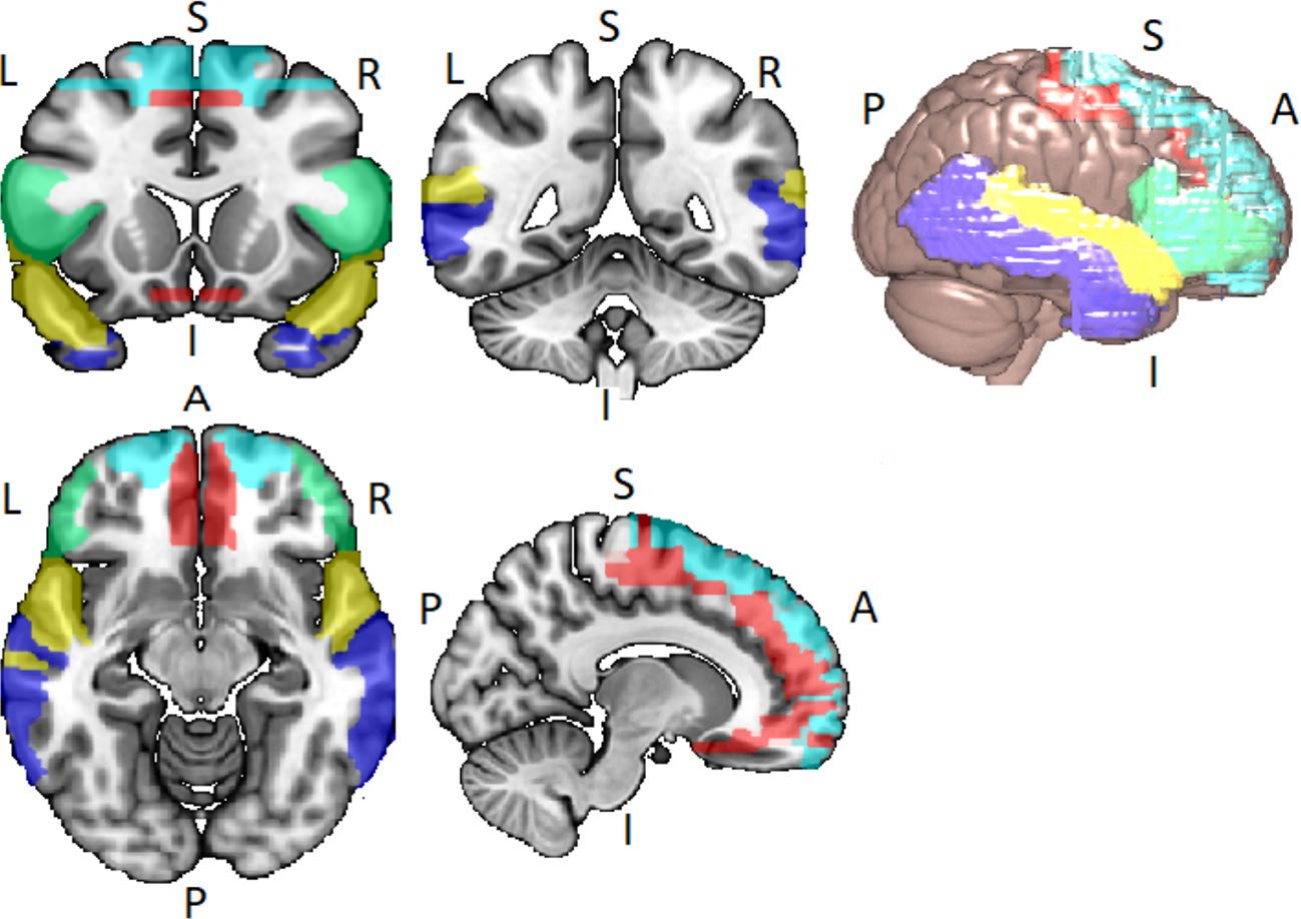
Lateral temporal and frontal network encompassing the inferior frontal gyrus (IFG) (green), the superior frontal gyrus (SFG) (light blue), the Middle Frontal Gyrus (MFG) (red), the superior temporal gyrus (STG) (yellow), and the middle temporal gyrus (MTG) (blue). Networks identified *via* WFU_PickAtlas toolbox for SPM12 in Montreal Neurological Insitute (MNI) space.

Disordered brain connectivity is thought to be a central pathophysiological feature of schizophrenia (72–75). The disconnection  hypothesis of  schizophrenia was initially motivated by neuroimaging studies showing abnormal patterns of functional connectivity between lateral temporal and frontal lobe regions (76–80). Lateral temporal and frontal lobe networks are important for a range of cognitive functions, particularly speech, thought, and language [see Ref. (81)]. The language network usually refers to the superior temporal gyrus (STG), middle temporal gyrus (MTG), the superior temporal sulcus (STS), the inferior frontal gyrus (IFG), frontal operculum, and adjacent regions in the inferior parietal lobe of both hemispheres, but with lateralization to the left hemisphere (82, 83) (see **Figure 2**).

Dysfunction in lateral temporal-centered networks in patients with schizophrenia is thought to underlie positive symptoms relating to language, thought, and speech such as auditory verbal hallucinations (10, 84–86) and thought disorder (87). To date, there are a limited number of studies suggesting altered function and connectivity in this network during prodromal stages of illness (22–24, 28). In a cross-sectional study, Crossley et al. (22) studied participants with CHR for psychosis, FEP patients, and healthy controls using a working memory task. Effective connectivity between frontal and temporal lobe regions was explicitly examined. There were differences in effective connectivity between the STG and prefrontal regions across the three groups, with a negative coupling between these areas in controls, a positive coupling in the FEP group, and intermediate effective connectivity parameters seen in the CHR group. In both the FEP and CHR groups, altered effective connectivity was accompanied by increased task-related STG activity. The authors concluded that a failure to deactivate the STG during tasks that engage the prefrontal cortex is already evident at psychosis onset and may reflect a disruption of frontal and lateral temporal connectivity in psychosis. These connectivity changes, albeit to a lesser extent, were also seen in the CHR stage (22). Also using a cross-sectional study design, Allen and colleagues (75) used the Hayling Sentence Completion Task (HSCT) to examine frontal and lateral temporal lobe connectivity in a CHR group. During the HSCT, the CHR group did not differ from healthy controls in terms of frontal or temporal activation. However, there was greater anterior cingulate cortex (ACC) activity in the CHR participants during incongruent HSCT trials. Effective connectivity analysis revealed intact task-dependent modulation of frontal to temporal effective connectivity in the CHR group, although endogenous connection strength between the ACC and the MTG was increased relative to healthy controls. The authors concluded that frontal and temporal functional integration in CHR states is intact, but may depend on increased engagement of the ACC, an effect not observed in healthy controls.

Using a prospective design, Sabb and colleagues (23) investigated language processing and underlying neural networks associated with discourse processing in a CHR group. CHR participants and healthy controls underwent Functional magnetic Resonance Imaging (fMRI) while performing a naturalistic discourse-processing paradigm. CHR participants were followed for 24 months post fMRI scanning to assess symptom severity and social outcome. Relative to controls, CHR participants showed increased neural activity in a network of language-associated brain regions, including the medial prefrontal cortex bilaterally, left IFG and MTG, as well as the anterior cingulate cortex (ACC). Further, increased activity in the left IFG, STG, and caudate predicted subsequent transition to psychosis in CHR subjects. Within the CHR sample, severity of thought disorder at follow-up was positively correlated with signal change in the left IFG, superior frontal gyrus, and left MTG.

Colibazzi and colleagues (24) also use a prospective design with data-driven approaches to resting-state fMRI data to examine resting functional connectivity in a CHR group. Multivariate analyses revealed between-group differences in whole-brain connectivity patterns in bilateral temporal areas, mostly affecting functional connections to the thalamus. The study shows that the established functional connectivity abnormalities in temporal lobe areas, observed in schizophrenia patients, are also present in the CHR period, with aberrant connectivity of the temporal cortex predictive of transition to psychosis in this CHR cohort.

A more recent prospective study investigating broader network organizations in a CHR cohort identified abnormal connectivity (88) in a cerebello-thalamo-cortical circuit and abnormal modular connectome organization (28) in CHR cases that later converted to psychosis. While these studies did not specifically set out to examine lateral temporal lobe connectivity, Collin et al. (28) reported that the brain regions implicated in early-course schizophrenia, including the STG, were most abnormal in terms of connectivity. Together, these functional imaging studies provide evidence of altered lateral temporal lobe activity and connectivity in the prodromal phase of psychosis and that altered connectivity in temporal lobe areas predicts the later onset of FEP.

In addition to the functional studies discussed above, volumetric and diffusion tensor imaging (DTI) studies in CHR cohorts also report changes in frontal and temporal regions. In their cross-sectional analysis, Borgwardt and colleagues (21) report significant volumetric differences between CHR and healthy control groups in the left STG, as well as in the insula and cingulate gyrus. A prospective comparison of CHR participants based on their clinical outcomes at follow-up (approximately 2 years) revealed that those who later developed psychosis had less gray matter in the STG and frontal regions in the right insula and IFG than those who did not develop psychosis. These findings are in line with an earlier prospective volumetric study (50) that also reported that conversion to FEP in a CHR cohort was associated with reduced gray matter volume in lateral temporal and inferior frontal regions.

However, a later longitudinal volumetric study in a larger CHR cohort (89) reported that CHR subjects who developed psychosis demonstrated a greater rate of gray matter loss only in prefrontal cortex regions relative to CHR nonconverters and healthy subjects with no volumetric differences in temporal lobe regions. Diffusion tensor imaging (DTI) studies in CHR cohorts have also produced mixed results. A systematic review by Vijayakumar and colleagues (90) included 12 studies examining white matter connectivity changes in the CHR stage. The review reports that although the exact location of white matter abnormalities remains uncertain, altered white matter connections in frontal–lateral temporal and fronto-limbic pathways, including the superior longitudinal and uncinate fasciculus, cingulum and corpus callosum, appear to be implicated in the CHR stage.

In summary, there is evidence for altered lateral temporal activity and/or connectivity and reduced volume in people with a CHR for psychosis. Despite the mixed and negative findings regarding lateral temporal (STG) connectivity [i.e., Refs. (22, 75)], a recent large CHR study has implicated altered connectivity in lateral temporal region, particularly in the CHR cases that later convert to psychosis (28). Volumetric studies in CHR cohorts, while robustly reporting reduced gray matter volume in frontal regions, suggest that reduced volume in lateral temporal lobe may also predict those CHR cases that convert to psychosis. Studies of structural white matter connectivity using DTI do report altered white matter in pathways connecting lateral temporal and frontal regions in the CHR stage. Taken together, the limited evidence to date suggests that changes in lateral temporal volume, function, and connectivity are present in the CHR stage and may also predict conversion to psychosis (see section Discussion: Emerging Temporal Lobe Dysfunction in Clinical High-Risk Populations, Attenuated Symptoms, and Transition to Psychosis). However, much more work is needed to confirm these findings and to understand how changes in lateral temporal lobe-centered networks relate to APS in CHR cohorts, particularly disorganized speech and language symptoms.

## Discussion: Emerging Temporal Lobe Dysfunction in CHR Populations, Attenuated Symptoms, and Transition to Psychosis

Taken together, neuroimaging studies in CHR cohorts suggest that structural and functional changes are seen in both medial and lateral temporal lobe regions *and* related cortical and subcortical networks. We will now consider how these neurobiological changes relate to APS and how dysfunction in these networks is associated with conversion to psychosis.

### Are Different Attenuated Psychotic Symptoms Related to Dysfunction in Different Temporal Lobe Networks?

**Figure 3:**
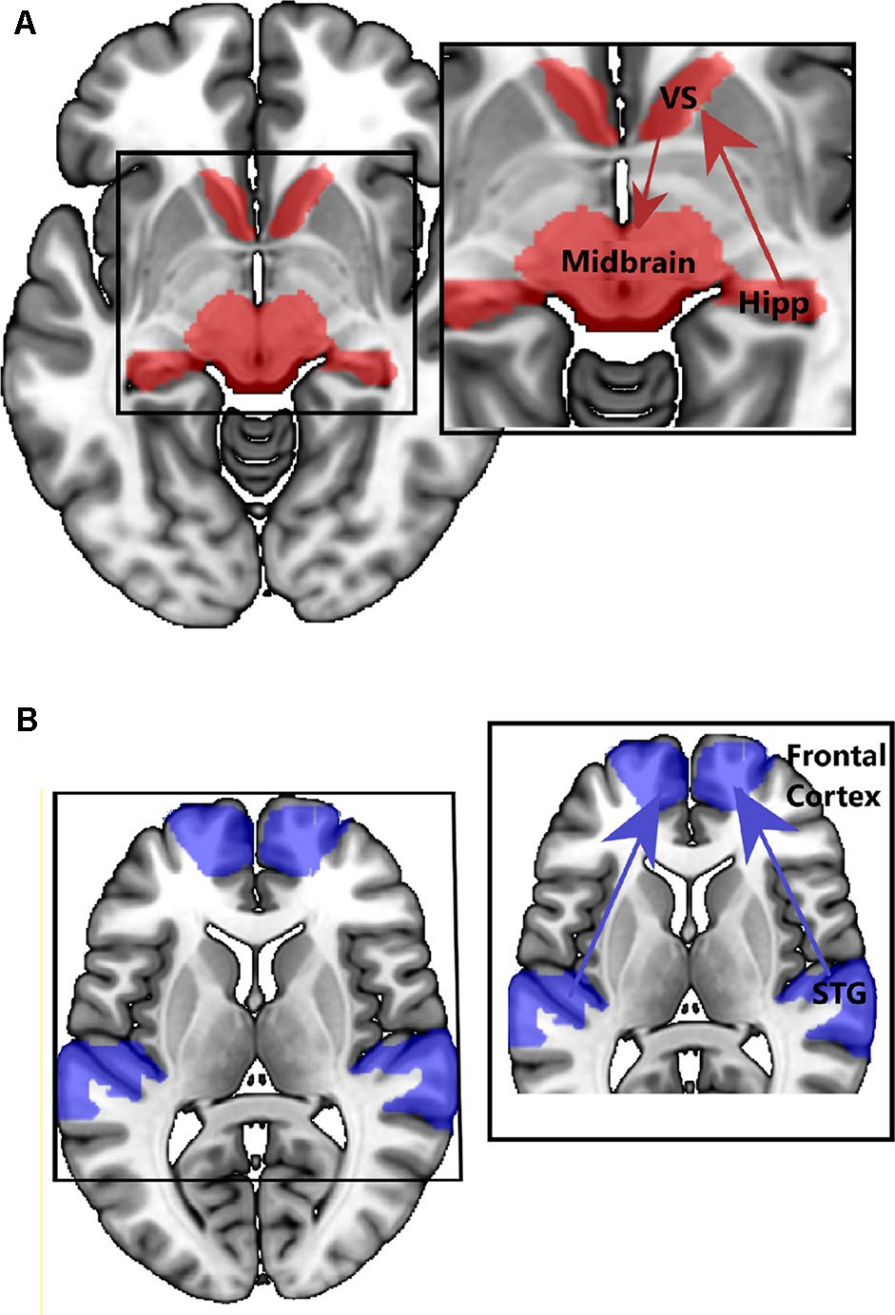
Example of connectivity findings in CHR samples. (**A**) Increased connectivity between hippocampus and striatum, and midbrain and striatum (Modinos, et al., 2019, 96). (**B**) Decreased connectivity between STG and prefrontal regions (89).

The studies discussed above point to emerging structural and functional alterations in medial and lateral temporal lobe networks in people at CHR for psychosis. So, are different (attenuated) psychotic symptoms in the CHR stage associated with dysfunction in different brain networks? One of the most influential neurobiological models of psychosis proposes that the inappropriate attribution of salience, to what would normally be irrelevant or neutral stimuli or experiences, underlies the formation of delusions (91). In support of this model, increased hippocampal activity is reported in CHR groups in response to neutral relative to emotional stimuli, suggesting impaired salience processing (92). Further, dopamine has a central role in mediating salience, and hyperdopaminergic states can lead to the aberrant assessment of salience (91, 93). To better understand the bio-behavioral significance of altered hippocampal neuronal activity in CHR cohorts, Modinos and colleagues (Modinos et al., 2019) used a novelty salience task in CHR participants and healthy controls. In CHR individuals, the hippocampal response to novel stimuli was significantly attenuated compared to that in the control group, possibly due to increased hippocampal activity at rest or in the control condition, consistent with previous findings of increased hippocampal rCBF (18, 45, 46). Modinos and colleagues also performed an effective connectivity analysis on these functional data and revealed that stimulus novelty (salience) modulated connections from the hippocampus to the ventral striatum significantly more in CHR participants than in controls, a finding seemingly consistent with the notion of increased hippocampal signaling/output to the striatum in psychosis (25). Conversely, stimulus novelty modulated connectivity from the midbrain (VTA) to the ventral striatum was significantly less in CHR participants than in HC, particularly in those CHR participants who subsequently developed psychosis at clinical follow-up. This pattern of activity and disconnectivity would be in line with the maximal tonic activation of dopamine neuron firing that is hypothesized to occur in psychosis, thought to obscure salience-driven increases in population activity of meso-striatal dopamine neurons (94) (see **Figure 3A**). A consequence of this might be the aberrant assignment of stimulus salience.

Previous neuroimaging studies of motivational and reward salience processing also suggest salience dysregulation and altered activation within a hippocampal–striatal–midbrain network in people at CHR for psychosis (95–97). Using a salience attribution task, Roiser and colleagues (97) found that CHR individuals were more likely to attribute motivational salience to irrelevant stimulus features and this bias was related to the severity of their delusion-like symptoms. Moreover, ventral striatal responses to irrelevant stimulus features were also correlated with delusion-like symptoms in CHR participants. Winton-Brown and colleagues (95) report that reward-induced modulation of connectivity from the ventral striatum/pallidum to the midbrain was greater in CHR participants than controls, and that in CHR participants, the strength of connectivity in this pathway is correlated with the severity of their abnormal beliefs. Collectively, these studies suggest that the behavioral consequence of emerging hippocampal–striatal–midbrain network dysregulation is aberrant salience processing that underlies delusional formation in the psychosis prodrome. Furthermore, these functional and connectivity findings in human CHR cohorts provide support for preclinical work positing that hippocampal hyperactivity (19, 26) results in perturbed striatal function (9, 25). This is important because taken together, these preclinical and human studies identify mechanisms and processes that lead to the development of psychotic symptoms, potentially identifying targets for intervention. Indeed, understanding the sequence of these pathological events, i.e., glutamatergic/GABAergic dysregulation, hippocampal hyperactivity, striatal dopamine dysregulation, that lead to aberrant salience and delusional formation, is crucial in the development and refinement of new treatments.

Disorganization in thought and language (37–42) is also observed in CHR individuals, and form part of the APS operational criterion for the assignment of a CHR state. Decades of neuroimaging research in people with schizophrenia have largely linked these symptoms to dysfunction, not in a hippocampal–striatal–midbrain network, but instead to altered function in lateral temporal regions and connectivity in lateral temporal, frontal, and parietal networks (**Figure 3B**). While there has been less research in CHR cohorts aimed at studying lateral temporal networks (23–28), their dysfunction, and relationship to symptoms, there are a large number of neuroimaging studies in established schizophrenia patients reporting that lateral temporal dysfunction and disconnectivity are associated with disorganized speech and language [see Ref. (87) for review] and auditory verbal hallucinations (10, 84, 85). It is tempting to speculate that these symptoms, ostensibly based around language and sensory dysfunction, have a different neurobiological basis than those relating to aberrant salience. Given that disorganized symptoms appear to predict conversion to FEP in CHR cohorts [e.g., Ref. (37)], from a biomarker perspective, it could be argued that dysfunction in lateral temporal lobe regions (and their associated networks) is a better predictor of conversion to FEP than hippocampal dysfunction. We will examine the evidence of this in the next section.

### Is Conversion to Psychosis Associated With Dysfunction in Just One or Both Temporal Networks?

At presentation, neuroimaging studies have identified a number of neurofunctional, neuroanatomical, and neurochemical changes in CHR cohorts relative to age-matched healthy controls. Furthermore, there is emerging evidence that neurobiological changes in temporal lobe regions at the CHR stage are directly related to APS. A small number of prospective and longitudinal neuroimaging studies have attempted to identify neural or biomarkers that predict conversion to psychosis in CHR cohorts. Multisite studies in CHR cohorts investigating volumetric changes associated with conversion to psychosis have reported different findings; one study reports that conversion to psychosis in CHR cohorts is associated with reduced left anterior hippocampal volume at presentation (15), while a second study reports that conversion is associated with reduced gray matter volume in prefrontal rather than temporal regions (89).

Functional MRI studies in CHR cohorts have reported that conversion to psychosis is associated with increased activation in the brainstem (midbrain/basilar pons) and the left hippocampus (10), the left STG, IFG, and caudate (23), and altered VTA–striatal effective connectivity (Modinos et al., 2019). PET studies using 18 Fluorodopa (18F-DOPA) also report increased striatal (12–14) and midbrain (10) presynaptic dopamine synthesis in CHR cases at presentation that later convert to psychosis. Two magnetic resonance spectroscopy (MRS) studies report increased striatal (98) and hippocampal (20) glutamate levels in CHR conversion cases, although another study reports that CHR individuals have significantly *lower* hippocampal Glx (combined glutamate and glutamine) levels relative to both healthy volunteers and FEP patients (99).

While these findings are broadly consistent with models that posit altered neurobiology in lateral/medial lobe regions and associated networks, not all prospective/longitudinal studies in CHR cohorts implicate the temporal lobes. For example, a recent study by Cao et al. (88) reports that CHR individuals show an intrinsic abnormality in *cerebellar–thalamic–frontal cortical* circuitry associated with disorganization symptoms that is significantly more pronounced among converters than nonconverters. Moreover, multivariate approaches to CHR outcome classification implicate a much wider range of cortical, cerebellar, and subcortical regions [e.g., Ref. (100)] making it difficult to attribute emergent APS to any specific network.

Together, these neuroimaging findings suggest that conversion to psychosis in CHR cohorts is associated with a wide range of structural, functional, and neurochemical alterations, i.e., altered structure and function in hippocampal, prefrontal, lateral temporal, midbrain, striatal, thalamic, and cerebellar regions as well as increased mesolimbic dopamine function and altered hippocampal and striatal glutamate/glutamine function. On the basis of these diverse neurobiological findings, it is difficult to attribute conversion to psychosis in CHR groups to a discrete region, network, or circuit. Moreover, there is some evidence from rodent models that disrupted hippocampal development can lead to neurophysiological abnormalities across several cortical regions, most prominently observed in prefrontal cortex circuits (66). We speculate, therefore, that hippocampal/MTL pathophysiology is seen early in development, but as the brain matures with this abnormality, other circuits, perhaps involving the lateral temporal and prefrontal cortex, become dysregulated. Currently, there are no studies that directly test this chronological prediction in humans although a cross-sectional study by Benetti and colleagues does link altered hippocampal and prefrontal function in CHR subject (101). Alternatively, in schizophrenia, there may be a pathological process playing out across multiple cortical and subcortical circuits, and the hippocampal–striatal–midbrain circuit may be where it is most evident (and possibly most severe) in the CHR/prodromal stage.

In summary, while there is evidence for emerging dysfunction in a range of cortical and subcortical regions in CHR cohorts, altered structure and function in medial and lateral temporal lobe regions, along with prefrontal regions, appear to be seen to a greater extent in CHR cases that convert to psychosis. While the evidence remains equivocal, several imaging studies adopting whole brain analytical approaches support this view [e.g., Refs. (10, 15, 21, 23, 24, 89)] and recent connectivity studies in large CHR data sets show that lateral temporal (28) and prefrontal cortex (88) disconnectivity are particularly associated with conversion to psychosis. Intriguingly, analysis of speech in CHR cohorts (a function strongly related to lateral temporal, frontal, and parietal lobe function) (81) shows that reduced semantic coherence and syntactic complexity predicted later psychosis development with 100% accuracy, outperforming classification from clinical interviews (102). These findings were cross-validated in a second larger cohort using a speech automated machine-learning speech classifier—comprising decreased semantic coherence, greater variance in that coherence, and reduced usage of possessive pronouns. This classifier had 83% accuracy in predicting psychosis onset (103). These findings are broadly in line with earlier factor analytical studies for CHR symptom dimensions that report disorganized thought and speech as predictor of subsequent conversion to psychosis (37). More generally, the neuropsychological data in CHR cohorts suggests that later conversion to psychosis is associated with marked deficits in verbal fluency, processing speed and memory domains (1), cognitive functions likely to rely on frontal, lateral temporal, and hippocampal activity and connectivity.

Based on the studies discussed here, several questions remain about emerging brain network dysfunction in CHR cohorts. Firstly, is dysfunction in both lateral and medial temporal networks necessary for the emergence of a CHR for psychosis or is dysfunction in one or the other network sufficient? Secondly, what is the pathological relationship between dysfunction in these temporal lobe networks? Third, do alterations in volume, function, and connectivity in these regions/networks occur at the same time, or is there a chronological progression related to illness stage, symptom severity, and risk (conversion) for psychosis? Fourth, is dysfunction in these networks related to different pathological or etiological processes?

Studies investigating schizotypal personality disorder (SPD) might provide preliminary insight regarding the first question, as SPD is conceded to represent an intermediate schizophrenia-spectrum phenotype (104). Evidence for temporal and frontal lobe alterations appears to be fairly robust in SPD samples. Similar to findings in CHR samples [e.g., Ref. (90)], investigations utilizing DTI in SPD populations report reduced fractional anisotropy in the uncinated fasciculus (but not in the cingulum) (105), reduced frontal and temporal lobe gray matter (106–109), and reduced temporal lobe gray matter thickness (106). Probabilistic tractography approaches also indicate that healthy individuals higher on psychometric schizotypy traits present with lower fractional anisotropy in the inferior fronto-occipital fasciculus overall (106). A recent fMRI study in an SPD population, investigating symptom clusters, reported that higher resting-state functional connectivity between the frontal and lateral temporal cortex was linked to positive and disorganized dimensions, respectively (110).

However, in SPD populations, there appears to be less evidence in line with rodent models of psychosis and findings in CHR cohorts. One recent study has shown that participants that score highly on measures of schizotypal personality traits (healthy participants with psychotic-like experiences) show higher rCBF in the right hippocampus compared to low-scoring individuals (111), although no differences were observed in midbrain or striatum. However, results from one early single-photon emission computed tomography (SPECT) study suggested relatively greater presynaptic dopamine release in the striatum of SPD individuals relative to healthy controls, similar to levels observed in patients with remitted schizophrenia (112).

Taken together, studies in SPD cohorts seem to provide more evidence for lateral temporal and frontal lobe dysfunction than they do for dysfunction in a hippocampal–striatal–midbrain circuit, although this may be because far less research has been conducted that explicitly examines the hippocampal–striatal–midbrain circuit in psychometric schizotypy/SPD populations (110–113).

Regarding the final question about etiology, there are some interesting clues from previous research. Although increased striatal dopamine function has been reported in CHR cohorts (12–14, 114), no increase in striatal dopamine synthesis was seen in a cohort of nonclinical voice hearers (114). However, increased lateral temporal activity while experiencing voices has been reported in nonclinical voice hearers (115). This finding suggests that dysfunction in the STG and wider frontal and temporal networks, which underlies the experience of auditory verbal hallucinations (10), is not necessarily related to elevated striatal dopamine function, suggesting the possibility of a different etiological pathway. However, it should be noted that a recent PET study by Cassidy et al. (116) reported a relationship between striatal dopamine release and auditory perceptual disturbances and outlined a novel dopamine-dependent mechanism for perceptual modulation that may confer vulnerability to hallucinations in hyperdopaminergic states underlying psychosis.

## Conclusions and Future Work

The literature discussed here strongly suggests that altered structure, function, and connectivity in both the medial and lateral temporal lobe play a key role in the development of CHR states and psychosis. Critically, increased rCBF and neural activity in the hippocampus seems to be a well-established factor in CHR populations, suggesting that abnormalities are present at the earliest stages of the condition. Further, studies have linked increased hippocampal glutamate and rCBF with conversion to psychosis, in line with rodent models suggesting that abnormalities in the hippocampus lead to dopamine dysfunctions and abnormal salience processing. While the etiology behind these hippocampal changes is not clear at this stage, immune/inflammatory responses appear to be a factor (117, 118).

Studies also report functional and volumetric abnormalities in the lateral temporal lobe and associated frontal–temporal and parietal language networks, possibly linked to the disorganized language and speech symptoms during the CHR stage. However, while disorganized speech and language symptoms appear to be a strong predictor of conversion to FEP in CHR cohorts, supported by recent findings using speech algorithms, findings from neuroimaging studies are far less equivocal, i.e., there is no clear evidence that lateral temporal lobe-centered networks are the best predictor of conversion to FEP.

While the findings discussed here strongly implicate hippocampal, lateral temporal lobe, and broader network dysfunction in CHR samples, the specific mechanisms or chronology of emerging brain network dysfunction in CHR cohorts remains unclear. Hippocampal abnormalities (leading to dopamine dysregulation) could lead to widespread abnormalities in other regions, particularly other (local) temporal lobe regions, which then lead to psychosis conversion. Alternatively, psychosis progression might be linked to widespread neural circuit abnormalities (with hippocampal dysfunction being most prominent). More sophisticated multimodal longitudinal studies are needed to test these alternative possibilities.

In addition to more sophisticated mechanistic studies, those utilizing novel pharmacological agents could provide valuable insights into whether potentially blocking increases in extracellular glutamate in MTL regions can ameliorate hypermetabolism and atrophy in CHR cohorts. A recent rodent study reported that glutamate dysregulation in the CA1 region of the hippocampus initiated the transition to syndromal psychosis (19). Investigating whether the administration of glutamate regulating drugs in CHR samples would normalize hippocampal hypermetabolism and prevent symptomatic and pathological progression may help to elucidate the mechanistic chronology during emerging psychosis.

Finally, it needs to be noted that schizophrenia is symptomatically heterogeneous and that symptoms and clinical outcomes in prodromal cases are even more heterogeneous. This may be because dysfunction in different neural circuits and neurotransmitter systems may predominate in different subtypes and stages of the disease, which, as defined, represents only a final common syndrome.

## Author Contributions

All authors listed have made substantial, direct, and intellectual contribution to the work and approved it for publication.

## Conflict of Interest Statement

The authors declare that the research was conducted in the absence of any commercial or financial relationships that could be construed as a potential conflict of interest.
